# Gibbs manifolds

**DOI:** 10.1007/s41884-023-00111-2

**Published:** 2023-07-03

**Authors:** Dmitrii Pavlov, Bernd Sturmfels, Simon Telen

**Affiliations:** 1 MPI-MiS, Leipzig, Germany; 2 UC Berkeley, Berkeley, USA; 3 CWI, Amsterdam, The Netherlands

**Keywords:** Gibbs variety, Toric geometry, Semidefinite programming, Quantum optimal transport, 68W30, 14M25, 90C22

## Abstract

Gibbs manifolds are images of affine spaces of symmetric matrices under the exponential map. They arise in applications such as optimization, statistics and quantum physics, where they extend the ubiquitous role of toric geometry. The Gibbs variety is the zero locus of all polynomials that vanish on the Gibbs manifold. We compute these polynomials and show that the Gibbs variety is low-dimensional. Our theory is applied to a wide range of scenarios, including matrix pencils and quantum optimal transport.

## Introduction

Toric varieties provide the geometric foundations for many successes in the mathematical sciences. In statistics they appear as discrete exponential families [27, p. 2], and their ideals reveal Markov bases for sampling from conditional distributions [5]. In optimization, they furnish nonnegativity certificates [9] and they govern the entropic regularization of linear programming [26]. Notable sightings in phylogenetics, stochastic analysis, Gaussian inference and chemical reaction networks gave us the slogan that *the world is toric* [19, Section 8.3].

In all of these applications, the key player is the positive part of the toric variety. That real manifold is identified with a convex polytope by the moment map [19, Theorem 8.24]. The fibers of the underlying linear map are polytopes of complementary dimension, and each fiber intersects the toric variety uniquely, in the *Birch point*. This is the unique maximizer of the entropy over the polytope [22, Theorem 1.10]. In statistical physics and computer science [29], the Birch point is known as the *Gibbs distribution*. The name Gibbs refers to the maximum entropy state in a quantum system, and this is also the reason behind our title.

This paper initiates a non-commutative extension of applied toric geometry. In that extension, points in $\mathbb {R}^n$ are replaced by real symmetric $n \times n$ matrices, and linear programming is replaced by semidefinite programming. There is a moment map which takes the cone of positive semidefinite matrices onto a spectrahedral shadow, and whose fibers are spectrahedra of complementary dimension. The Gibbs manifold plays the role of the positive toric variety. Each spectrahedron intersects the Gibbs manifold uniquely, in the *Gibbs point*. Just like in the toric case, we study these objects algebraically by passing to the Zariski closure of our positive manifold. The resulting analogues of toric varieties are called *Gibbs varieties*.

We illustrate these concepts for the following linear space of symmetric $3 \times 3$-matrices:

1 $$\begin{aligned} {\mathcal {L}} \,\,\, =\,\,\, \left\{ \, \begin{bmatrix} y_1+y_2+y_3 &{} y_1 &{} y_2 \\ y_1 &{} y_1+y_2+y_3 &{} y_3 \\ y_2 &{} y_3 &{} y_1+y_2+y_3 \end{bmatrix} : \,\, y_1,y_2,y_3 \in \mathbb {R}\, \right\} . \end{aligned}$$

The Gibbs manifold $\textrm{GM}({\mathcal {L}})$ is obtained by applying the exponential function to each matrix in ${\mathcal {L}}$. Since the matrix logarithm is the inverse to the matrix exponential, it is a 3-dimensional manifold, contained in the 6-dimensional cone $\textrm{int}({\mathbb {S}}^3_+)$ of positive definite $3 \times 3$ matrices.

The quotient map from the matrix space ${\mathbb {S}}^3 \simeq \mathbb {R}^6$ onto ${\mathbb {S}}^3/{\mathcal {L}}^\perp \simeq \mathbb {R}^3$ takes positive semidefinite matrices $X = [x_{ij}]$ to their inner products with the matrices in a basis of ${\mathcal {L}}$:

$$\begin{aligned} \pi : \,{\mathbb {S}}^3_+ \rightarrow \mathbb {R}^3 \,:\, X \, \mapsto \, \bigl (\,\textrm{trace}(X) + 2x_{12},\, \textrm{trace}(X) + 2x_{13}, \,\textrm{trace}(X) + 2x_{23} \,\bigr ). \end{aligned}$$

Precisely this map appeared in the statistical study of Gaussian models in [25, Example 1]. The fibers $\pi ^{-1}(b)$ are three-dimensional spectrahedra, and these serve as feasible regions in optimization, both for semidefinite programming and for maximum likelihood estimation.

We here consider yet another convex optimization problem over the spectrahedron $\pi ^{-1}(b)$, namely maximizing the *von Neumann entropy* $\, h(X) = \textrm{trace}(X- X \cdot \textrm{log}(X))$. This problem has a unique local and global maximum, at the intersection $\pi ^{-1}(b) \,\cap \,\mathrm{GM({\mathcal {L}})}$. See Theorem 5.1. This *Gibbs point* is the maximizer of the entropy over the spectrahedron. Therefore, the Gibbs manifold $\textrm{GM}({\mathcal {L}})$ is the set of Gibbs points in all fibers $\pi ^{-1}(b)$, as *b* ranges over $\mathbb {R}^3$.

To study these objects algebraically, we ask for the polynomials that vanish on $\textrm{GM}({\mathcal {L}})$. The zeros of these polynomials form the *Gibbs variety* $\textrm{GV}({\mathcal {L}})$. Thus, the Gibbs variety is the Zariski closure of the Gibbs manifold. In our example, the Gibbs manifold has dimension 3, whereas the Gibbs variety has dimension 5. The latter is the cubic hypersurface

$$\begin{aligned} \textrm{GV}({\mathcal {L}}) \,= \,{} & {} \bigl \{X \in {\mathbb {S}}^3: (x_{11}-x_{22})(x_{11}-x_{33})(x_{22}-x_{33}) \\{} & {} \quad = x_{33}(x_{13}^2-x_{23}^2) + x_{22}(x_{23}^2-x_{12}^2) + x_{11}(x_{12}^2 -x_{13}^2) \bigr \}. \end{aligned}$$

As promised, the study of Gibbs manifolds and Gibbs varieties is a non-commutative extension of applied toric geometry. Indeed, every toric variety is a Gibbs variety arising from diagonal matrices. For instance, the toric surface $\,\{ \,x \in \mathbb {R}^3: x_1 x_3 \,=\, x_2^2\, \}\,$ is realized as

$$\begin{aligned} \textrm{GV}({\mathcal {L}}') \, = \, \bigl \{X \in {\mathbb {S}}^3:\, x_{11} x_{33} - x_{22}^2 \, = \, x_{12} = x_{13} = x_{23} = 0 \,\bigr \} \end{aligned}$$

for the diagonal matrix pencil

2 $$\begin{aligned} {\mathcal {L}}' \, = \, \left\{ \, \begin{bmatrix} 2 y_1 &{} 0 &{} 0 \\ 0 &{} y_1{+}y_2 &{} 0 \\ 0 &{} 0 &{} 2 y_2 \end{bmatrix} : \,\, y_1,y_2 \in \mathbb {R}\, \right\} . \end{aligned}$$

However, even for diagonal matrices, the dimension of the Gibbs variety can exceed that of the Gibbs manifold. To see this, replace the matrix entry $2 y_1$ by $\sqrt{2} y_1$ in the definition of ${\mathcal {L}}'$. This explains why transcendental number theory will make an appearance in this work.

Our presentation in this paper is organized as follows. Section 2 gives a more thorough introduction to Gibbs manifolds and Gibbs varieties. Theorem 2.4 states that the dimension of the Gibbs variety is usually quite small. The proof of this result is presented in Sect. 3. In that section we present algorithms for computing the prime ideal of the Gibbs variety. This is an implicitization problem, where the parametrization uses transcendental functions. We compare exact symbolic methods for solving that problem with a numerical approach. A key ingredient is the Galois group for the eigenvalues of a linear space of symmetric matrices. We implemented our algorithms in Julia, making use of the computer algebra package Oscar.jl [21]. Our code and data are available at https://mathrepo.mis.mpg.de/GibbsManifolds.

In Sect. 4 we study the Gibbs varieties given by two-dimensional spaces of symmetric matrices. This rests on the classical Segre-Kronecker classification of matrix pencils [8].

In Sect. 5 we turn to the application that led us to start this project, namely *entropic regularization* in convex optimization. That section develops the natural generalization of the geometric results in [26] from linear programming to semidefinite programming. We conclude in Sect. 6 with a study of *quantum optimal transport* [3]. This is the semidefinite programming analogue to the classical optimal transport problem [26, Section 3]. We show that its Gibbs manifold is the positive part of a Segre variety in matrix space.

## From manifolds to varieties

We write ${\mathbb {S}}^n$ for the space of symmetric $n \times n$-matrices. This is a real vector space of dimension $$\left( {\begin{array}{c}n+1\\ 2\end{array}}\right) $$. The subset of positive semidefinite matrices is denoted ${\mathbb {S}}^n_+$. This is a full-dimensional closed semialgebraic convex cone in ${\mathbb {S}}^n$, known as the *PSD cone*. The PSD cone is self-dual with respect to the trace inner product, given by $\, \langle X, Y \rangle \,:=\, \textrm{trace}(X Y)$ for $X, Y \in {\mathbb {S}}^n$.

The matrix exponential function is defined by the usual power series, which converges for all real and complex $n \times n$ matrices. It maps symmetric matrices to positive definite symmetric matrices. The zero matrix $0_n$ is mapped to the identity matrix $\textrm{id}_n$. We write

$$\begin{aligned} \textrm{exp} :\, {\mathbb {S}}^n \rightarrow \textrm{int} ({\mathbb {S}}^n_+), \,\, X \,\mapsto \, \sum _{i=0}^\infty \, \frac{1}{i !} \,X^i. \end{aligned}$$

This map is invertible, with the inverse given by the familiar series for the logarithm, which is convergent for any positive definite matrix:

$$\begin{aligned} \textrm{log}\,:\, \textrm{int} ({\mathbb {S}}^n_+) \rightarrow {\mathbb {S}}^n,\,\, Y \,\mapsto \, \sum _{j=1}^\infty \frac{(-1)^{j-1}}{j} \,( \,Y - \textrm{id}_n)^j. \end{aligned}$$

We next introduce the geometric objects studied in this article. We fix any matrix $A_0 \in {\mathbb {S}}^n$ and *d* linearly independent matrices $A_1, A_2,\ldots ,A_d$, also in ${\mathbb {S}}^n$. We write ${\mathcal {L}}$ for the affine subspace $\,A_0 + \textrm{span}_{{\mathbb {R}}}(A_1, \ldots , A_d)\,$ of the vector space $${\mathbb {S}}^n \simeq \mathbb {R}^{\left( {\begin{array}{c}n+1\\ 2\end{array}}\right) }$$. Thus, ${\mathcal {L}}$ is an *affine space of symmetric matrices* (ASSM) of dimension *d*. If $A_0 = 0$, then ${{{\mathcal {L}}}}$ is a *linear space of symmetric matrices* (LSSM). We are interested in the image of ${{{\mathcal {L}}}}$ under the exponential map:

### Definition 2.1

The *Gibbs manifold* $\textrm{GM}({{{\mathcal {L}}}})$ of ${\mathcal {L}}$ is the *d*-dimensional manifold $\textrm{exp}({\mathcal {L}}) \subset {\mathbb {S}}^n_+$.

This is indeed a *d*-dimensional manifold inside the convex cone ${\mathbb {S}}^n_+$. It is diffeomorphic to ${\mathcal {L}} \simeq \mathbb {R}^d$, with the identification given by the exponential map and the logarithm map.

In notable special cases (e.g. that in Sect. 6), the Gibbs manifold is semi-algebraic, namely it is the intersection of an algebraic variety with the PSD cone. However, this fails in general, as seen in the Introduction. It is still interesting to ask which polynomial relations hold between the entries of any matrix in $\textrm{GM}({{{\mathcal {L}}}})$. This motivates the following definition.

### Definition 2.2

The *Gibbs variety* $\textrm{GV}({{{\mathcal {L}}}})$ of ${\mathcal {L}}$ is the Zariski closure of $\textrm{GM}({{{\mathcal {L}}}})$ in $${\mathbb {C}}^{\left( {\begin{array}{c}n+1\\ 2\end{array}}\right) }$$.

### Example 2.3

($n = 4, d = 2$) Consider the 2-dimensional linear space of symmetric matrices

$$\begin{aligned} {{{\mathcal {L}}}} \,\,=\,\, \left\{ \, \begin{bmatrix} 0 &{} 0 &{} 0 &{} y_1 \\ 0 &{} 0 &{} y_1 &{} y_2 \\ 0 &{} y_1 &{} y_2 &{} 0 \\ y_1 &{} y_2 &{} 0 &{} 0 \end{bmatrix}:\, \, y_1, y_2 \in {\mathbb {R}} \, \right\} \, \,\subset \,\, {\mathbb {S}}^4. \end{aligned}$$

Its Gibbs manifold $\textrm{GM}({{{\mathcal {L}}}})$ is a surface in ${\mathbb {S}}^4 \simeq {\mathbb {R}}^{10}$. The Gibbs variety $\textrm{GV}({{{\mathcal {L}}}})$ has dimension five and degree three. It consists of all symmetric matrices $X = (x_{ij})$ whose entries satisfy

3 $$\begin{aligned} \begin{matrix} \qquad x_{13}-x_{22}+x_{44} \,= \,x_{14}-x_{23}+x_{34} \,=\, \,x_{24}-x_{33}+x_{44} \,=\, 0, \\ \textrm{and} \qquad \textrm{rank} \begin{bmatrix} x_{11}{-}x_{44} &{} x_{12}{-}x_{34} &{} x_{22}{-}x_{33} \\ x_{12} &{} x_{22}{-}x_{44} &{} x_{23}{-}x_{34} \end{bmatrix} \,\le \,1. \qquad \end{matrix} \end{aligned}$$

This follows from the general result on matrix pencils in Theorem 4.4.$\diamond$

The following dimension bounds constitute our main result on Gibbs varieties.

### Theorem 2.4

Let ${{{\mathcal {L}}}} \subset {\mathbb {S}}^n$ be an ASSM of dimension *d*. The dimension of the Gibbs variety $\textrm{GV}({{{\mathcal {L}}}})$ is at most $n + d$. If $A_0 = 0$, i.e. ${{{\mathcal {L}}}}$ is an LSSM, then $\textrm{dim} \, \textrm{GV}({{{\mathcal {L}}}})$ is at most $n + d-1$.

These bounds are attained in many cases, including Example 2.3. Our proof of Theorem 2.4 appears in Sect. 3, in the context of algorithms for computing the ideal of $\textrm{GV}({{{\mathcal {L}}}})$.

While it might be difficult to find all polynomials that vanish on the Gibbs manifold, finding *linear* relations is sometimes easier. Such relations are useful for semidefinite optimization, see Remark 5.2. This brings us to the final geometric object studied in this paper.

### Definition 2.5

The *Gibbs plane* $\textrm{GP}({{{\mathcal {L}}}})$ is the smallest affine space containing $\textrm{GV}({{{\mathcal {L}}}})$.

Clearly, we have the chain of inclusions $$\,\textrm{GM}({{{\mathcal {L}}}}) \,\subseteq \,\textrm{GV}({{{\mathcal {L}}}}) \,\subseteq \, \textrm{GP}({{{\mathcal {L}}}}) \,\subseteq \, {\mathbb {C}}^{\left( {\begin{array}{c}n+1\\ 2\end{array}}\right) }$$.

### Example 2.6

The Gibbs plane of the LSSM ${{{\mathcal {L}}}}$ from Example 2.3 is the 7-dimensional linear space in $\mathbb {C} ^{10}$ that is defined by the three linear relations listed in the first row of (3). $\diamond$

It was claimed in the Introduction that this article offers a generalization of toric varieties. We now make that claim precise, by discussing the case when ${{{\mathcal {L}}}}$ is a commuting family. This means that the symmetric matrices $A_0, A_1, \ldots , A_d$ commute pairwise, i.e. $A_i A_j = A_j A_i$ for all *i*, *j*. We now assume that this holds. Then the ASSM ${\mathcal {L}}$ can be diagonalized [14, Theorem 1.3.19], i.e. there is an orthogonal matrix *V* such that $\Lambda _i = V^\top A_iV$ is a diagonal matrix, for all *i*. The vector $\lambda _i \in {\mathbb {R}}^n$ of diagonal entries in $\Lambda _i = \textrm{diag}(\lambda _i)$ contains the eigenvalues of $A_i$.

The matrix exponential of any element in ${{{\mathcal {L}}}}$ can be computed as follows:

4 $$\begin{aligned} \textrm{exp}(A_0 + y_1A_1 + \cdots + y_d A_d)= V \cdot \textrm{exp}(\Lambda _0 + y_1 \Lambda _1 + \cdots + y_d \Lambda _d) \cdot V^\top . \end{aligned}$$

Let ${\mathcal {D}}$ denote this ASSM of diagonal matrices, i.e. $\,{\mathcal {D}} \,= \, \{ \Lambda _0 + y_1 \Lambda _1 + \cdots + y_d \Lambda _d \,:\, y \in \mathbb {R}^d \}$. Then the linear change of coordinates given by *V* identifies the respective Gibbs manifolds:

5 $$\begin{aligned} \textrm{GM}({\mathcal {L}}) \, = \, V \cdot \textrm{GM}({\mathcal {D}}) \cdot V^\top . \end{aligned}$$

The same statement holds for the Gibbs varieties and the Gibbs planes:

6 $$\begin{aligned} \textrm{GV}({\mathcal {L}}) \, = \, V \cdot \textrm{GV}({\mathcal {D}}) \cdot V^\top \qquad \textrm{and} \qquad \textrm{GP}({\mathcal {L}}) \, = \, V \cdot \textrm{GP}({\mathcal {D}}) \cdot V^\top . \end{aligned}$$

The dimensions of these objects are determined by arithmetic properties of the eigenvalues.

We identify the space of diagonal $n \times n$-matrices with ${\mathbb {R}}^n$. Recall that $\Lambda _i = \textrm{diag}(\lambda _i)$ where $\lambda _i$ is a vector in $\mathbb {R}^n$. Let $\Lambda$ denote the linear subspace of $\mathbb {R}^n$ that is spanned by the *d* vectors $\,\lambda _1,\ldots ,\lambda _d$. We have ${\mathcal {D}} = \lambda _0 + \Lambda$, and therefore

$$\begin{aligned} \textrm{GM}({\mathcal {D}}) \,\, = \,\, \textrm{exp}(\lambda _0) \, \star \, \textrm{exp}(\Lambda ) \, = \, \{ (e^{\lambda _{01}} w_1, \ldots , e^{\lambda _{0n}} w_n) : \, w \in \textrm{exp}(\Lambda ) \} \,\,\, \subset \,\,\, \mathbb {R}^n. \end{aligned}$$

Here $\star$ denotes coordinate-wise multiplication in $\mathbb {R}^n$. Let $\Lambda _\mathbb {Q}$ be the smallest vector subspace of $\mathbb {R}^n$ spanned by elements of $\mathbb {Q}^n$ which contains $\Lambda$. Its dimension $\,d_\mathbb {Q}= \textrm{dim}\,\Lambda _\mathbb {Q}\,$ satisfies $\, d \le d_\mathbb {Q}\le n$. Fix lattice vectors $\,a_1,a_2,\ldots ,a_{d_\mathbb {Q}}\,$ in $\mathbb {Z}^n$ that form a basis of $\Lambda _\mathbb {Q}$. Then, inside the *n*-dimensional linear space of diagonal matrices, we have

7 $$\begin{aligned} \textrm{GV}({\mathcal {D}}) =\overline{\biggl \{\bigl (\, e^{\lambda _{01}} \prod _{i=1}^{d_\mathbb {Q}} z_i^{a_{i1}},\,\, e^{\lambda _{02}} \prod _{i=1}^{d_\mathbb {Q}} z_i^{a_{i2}},\,\ldots ,\,\, e^{\lambda _{0n}} \prod _{i=1}^{d_\mathbb {Q}} z_i^{a_{in}}\, \bigr ) :\,\, z \in (\mathbb {C} ^{*})^{d_\mathbb {Q}} \biggr \}}. \end{aligned}$$

This is a toric variety of dimension $d_\mathbb {Q}$. Just like in [26, Section 2], the closure is taken in $\mathbb {C} ^n$. The Gibbs manifold $\textrm{GM}({\mathcal {D}})$ is a *d*-dimensional subset of the real points in $\textrm{GV}({\mathcal {D}})$ for which *z* has strictly positive coordinates. We summarize our discussion in the following theorem.

### Theorem 2.7

Let ${\mathcal {L}}$ be an affine space of pairwise commuting symmetric matrices. Then the Gibbs variety $\textrm{GV}({\mathcal {L}})$ is a toric variety of dimension $d_\mathbb {Q}$, given explicitly by (6) and (7).

For an illustration, consider the seemingly simple case $d=1$ and $A_0 = 0$. Here, $\textrm{GM}({\mathcal {L}})$ is the curve formed by all powers of $\textrm{exp}(A_1)$, and $\textrm{GV}({\mathcal {L}})$ is a toric variety of generally higher dimension. This scenario is reminiscent of that studied by Galuppi and Stanojkovski in [10].

### Example 2.8

Let $n=3$ and consider the LSSM ${\mathcal {L}}$ spanned by $$ A_1 = \begin{bmatrix} 4 &{} 1 &{} 1 \\ 1 &{} 3 &{} 1 \\ 1 &{} 1 &{} 3 \end{bmatrix}$$. We have

$$\begin{aligned} A_1 \,= & {} \, V \cdot \textrm{diag} (\lambda ) \cdot V^\top , \,\, \textrm{where} \quad \lambda = \bigl (2, 4 + \sqrt{2}, 4 - \sqrt{2} \bigr ) \quad \textrm{and} \quad \end{aligned}$$

$$\begin{aligned} V \,= & {} \, \frac{1}{2} \begin{bmatrix} 0 &{} \sqrt{2} &{} - \sqrt{2} \\ - \sqrt{2} &{} 1 &{} 1 \\ \sqrt{2} &{} 1 &{} 1 \end{bmatrix}. \end{aligned}$$

Here, ${\mathcal {D}} = \Lambda = \mathbb {R}\lambda$, $\,d_\mathbb {Q}= 2$, and $\Lambda _\mathbb {Q}\,=\,\mathbb {R}\{ (1,2,2), (0,1,-1) \} \,=\, \{p \in \mathbb {R}^3 \,:\, 4 p_1 = p_2 + p_3 \}$. Hence $\textrm{GV}({\mathcal {D}})$ is the toric surface $\{q_{11}^4 = q_{22} q_{33}\}$ in $\,\textrm{GP}({\mathcal {D}}) = \{ Q \in {\mathbb {S}}^3\,:\, q_{12} = q_{13} = q_{23} = 0 \}$. We transform that surface into the original coordinates via (6). The computation reveals

$$\begin{aligned} \textrm{GV}({\mathcal {L}})= & {} \{ X \in \textrm{GP}({\mathcal {L}}):\,x_{23}^4-4 x_{23}^3x_{33} +6x_{23}^2 x_{33}^2-4 x_{23} x_{33}^3+x_{33}^4\\{} & {} +2 x_{13}^2-x_{23}^2-2x_{23}x_{33}-x_{33}^2 = 0 \}. \end{aligned}$$

The ambient 3-space is $\, \textrm{GP}({\mathcal {L}}) = \{ X \in {\mathbb {S}}^3: x_{11}-x_{23}-x_{33} = x_{12}-x_{13} =x_{22}-x_{33} = 0\}$.$\diamond$

This concludes our discussion of the toric Gibbs varieties that arise from pairwise commuting matrices. In the next section we turn to the general case, which requires new ideas.

## Implicitization of Gibbs varieties

Implicitization is the computational problem of finding implicit equations for an object that comes in the form of a parametrization. When the parametrizing functions are rational functions, these equations are polynomials and can be found using resultants or Gröbner bases [19, Section 4.2]. A different approach rests on polynomial interpolation and numerical nonlinear algebra. This section studies the implicitization problem for Gibbs varieties. The difficulty arises from the fact that Gibbs manifolds are transcendental, since their parametrizations involve the exponential function. We start out by presenting our proof of Theorem 2.4.

As in Sect. 2, ${{{\mathcal {L}}}} = A_0 + \textrm{span}_{{\mathbb {R}}}(A_1, \ldots , A_d)$ is a *d*-dimensional affine space of symmetric $n \times n$-matrices. Its elements are $A_0 + y_1A_1 + \cdots + y_d A_d$. We shall parametrize the Gibbs manifold $\textrm{GM}({{{\mathcal {L}}}})$ in terms of the coordinates $y_1, \ldots , y_d$ on ${{{\mathcal {L}}}}$. This uses the following formula.

### Theorem 3.1

(Sylvester [28]) Let $f: D \rightarrow {\mathbb {R}}$ be an analytic function on an open set $D \subset {\mathbb {R}}$ and $M \in {\mathbb {R}}^{n \times n}$ a matrix that has *n* distinct eigenvalues $\lambda _1,\ldots ,\lambda _n$ in *D*. Then

$$\begin{aligned} f(M) \,=\, \sum \limits _{i=1}^{n}f(\lambda _i)M_i, \quad \text {with} \quad M_i \,=\, \prod _{j\ne i}\dfrac{1}{\lambda _i-\lambda _j}(M - \lambda _j \cdot \textrm{id}_n). \end{aligned}$$

We note that the product on the right hand side takes place in the commutative ring $\,\mathbb {R}[M]$.

### Proof of Theorem 2.4

The characteristic polynomial of $A(y) = A_0 + y_1 A_1 + \cdots + y_d A_d$ equals

$$\begin{aligned} P_{{{\mathcal {L}}}}(\lambda ;y) \, = \textrm{det}(A(y) - \lambda \cdot \textrm{id}_n) \,=\, \, c_0(y) + c_1(y) \, \lambda + \cdots + c_{n-1}(y) \, \lambda ^{n-1} + (-1)^n \, \lambda ^n. \end{aligned}$$

Its zeros $\lambda$ are algebraic functions of the coordinates $y = (y_1, \ldots , y_d)$ on ${\mathcal {L}}$.

We first assume that ${\mathcal {L}}$ has distinct eigenvalues, i.e. there is a Zariski open subset $U \subset {\mathbb {R}}^d$ such that $P_{{{\mathcal {L}}}}(\lambda ;y^*)$ has *n* distinct real roots $\lambda$ for all $y^* \in U$. Sylvester’s formula writes the entries of $\textrm{exp}(A(y))$ as rational functions of $y, \lambda _i(y)$ and $e^{\lambda _i(y)}$ for $y \in U$. These functions are multisymmetric in the pairs $(\lambda _i, e^{\lambda _i})$. They evaluate to convergent power series on *U*.

Let *V* be the subvariety of $U \times {\mathbb {R}}^n$ that is defined by the equations

8 $$\begin{aligned} c_i(y) \, = \, (-1)^i \sigma _{n-i}(\lambda ) \quad \textrm{for} \quad i = 0, \ldots , n-1, \end{aligned}$$

where $(\lambda _1, \ldots , \lambda _n)$ are the coordinates on ${\mathbb {R}}^n$ and $\sigma _t(\lambda )$ is the $t^{\textrm{th}}$ elementary symmetric polynomial evaluated at $(\lambda _1, \ldots , \lambda _n)$. We have $\dim V = d$. Define a map $\phi : V \times {\mathbb {R}}^n \rightarrow {\mathbb {S}}^n$, using coordinates $z_1, \ldots , z_n$ on ${\mathbb {R}}^n$, as follows:

9 $$\begin{aligned} (y_1, \ldots , y_d, \lambda _1, \ldots , \lambda _n, z_1, \ldots , z_n) \, \longmapsto \, \sum _{i=1}^n z_i \, \prod _{j \ne i} \frac{1}{\lambda _i-\lambda _j} (A(y) - \lambda _j \cdot \textrm{id}_n). \end{aligned}$$

The closure $\overline{\phi (V \times {\mathbb {R}}^n)}$ of the image of this map is a variety. It contains the Gibbs variety: setting $z_i = e^{\lambda _i}$ parametrizes a dense subset of the Gibbs manifold, by Theorem 3.1.

The Gibbs variety of the LSSM $\mathbb {R}{{{\mathcal {L}}}}$ spanned by the ASSM ${{{\mathcal {L}}}}$ also lies in $\overline{\phi (V \times {\mathbb {R}}^n)}$, because $\textrm{exp}(y_0 A(y))=\phi (y_0 \cdot y, y_0 \cdot \lambda , e^{y_0 \cdot \lambda })$ for any $y \in U$ and $y_0 \in {\mathbb {R}} \backslash \{0\}$. We thus have

$$\begin{aligned} \dim \textrm{GV}({{{\mathcal {L}}}}) \,\le \,\dim \overline{\phi (V \times {\mathbb {R}}^n)}\, \le \,d + n \quad \text {and} \quad \dim \textrm{GV}( \mathbb {R}{{{\mathcal {L}}}})\, \le \,d + n. \end{aligned}$$

Finally, suppose that ${{{\mathcal {L}}}}$ is an LSSM, i.e. $A_0 = 0$. Then ${\mathcal {L}}$ is the linear span of an ASSM of dimension $d-1$ in ${\mathbb {S}}^n$. The second inequality therefore gives $\,\dim \textrm{GV}({{{\mathcal {L}}}}) \le d + n -1$.

We finally consider the case when ${{{\mathcal {L}}}}$ has $m < n$ distinct eigenvalues $\lambda _1,\ldots ,\lambda _m$ with multiplicities $\mu _1,\ldots ,\mu _m$. That is, there is a Zariski open subset $U \subset {\mathbb {R}}^d$ such that $P_{{{\mathcal {L}}}}(\lambda ;y^*)$ has *m* distinct real roots $\lambda$ with multiplicities $\mu _1,\ldots ,\mu _m$ for all $y^* \in U$. Since symmetric matrices are diagonalizable, Sylvester’s formula can easily be adapted to this case: it suffices to sum over the distinct eigenvalues of *M*, ignoring the multiplicities. That is, we replace *n* in the statement of Theorem 3.1 by *m*. See [15, Chapter 6.1, Problem 14(a)] for details. The variety *V* now lives in $U \times {\mathbb {R}}^m$. It is still defined by the equations (8) but now $\sigma _t(\lambda )$ is evaluated at $(\lambda _1,\ldots ,\lambda _1,\ldots ,\lambda _m,\ldots ,\lambda _m)$, where $\lambda _i$ appears $\mu _i$ times. The parametrization (9) takes the form of a map $\phi : V\times {\mathbb {R}}^m$ defined by (9) with every occurrence of *n* replaced by *m*. With this adjustments made, the proof repeats the case of *n* distinct eigenvalues. $\square$

### Remark 3.2

If the points $\textrm{exp}(\lambda (y)) = (e^{\lambda _1(y)}, \ldots , e^{\lambda _n(y)})$, $y \in U$, lie in a lower-dimensional subvariety $W \subset {\mathbb {R}}^n$, then the proof of Theorem 2.4 gives the better bound $\dim \textrm{GV}({{{\mathcal {L}}}}) \le d + \dim W$. We saw this in Example 2.8. In general, no such subvariety *W* exists, i.e. one expects $W = {\mathbb {R}}^n$. This is an issue of Galois theory, to be discussed at the end of this section.

For ease of exposition, we work only with LSSMs in the rest of this section. That is, we set $A_0 = 0$. We comment on the generalization to ASSMs in Remark 3.7. Our discussion and the proof of Theorem 2.4 suggest Algorithm 1, for computing the ideal of the Gibbs variety of an LSSM ${{{\mathcal {L}}}}$.

**Figure:**
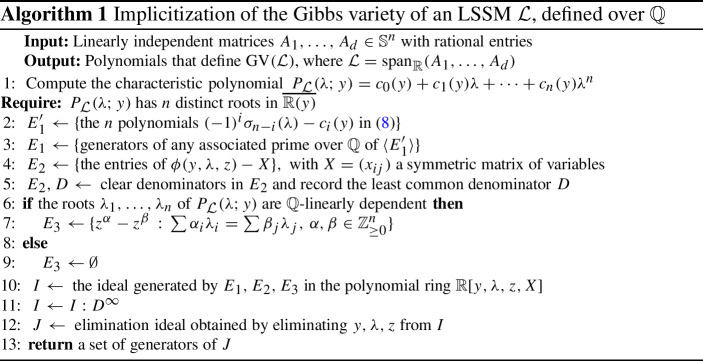
Implicitization of the Gibbs variety of an LSSM ${{{\mathcal {L}}}}$, defined over ${\mathbb {Q}}$

That ideal lives in a polynomial ring $\mathbb {R}[X]$ whose variables are the entries of a symmetric $n \times n$ matrix. The algorithm builds three subsets $E_1, E_2, E_3$ of the larger polynomial ring ${\mathbb {R}}[y,\lambda ,z, X]$. After the saturation (step 11), the auxiliary variables $y, \lambda , z$ are eliminated. The equations $E_1'$ come from (8). They constrain $(y,\lambda )$ to lie in *V*. The set $E_1$ generates an associated prime of $\langle E_1' \rangle$ (step 3), see the discussion preceding Theorem 3.6. The equations $E_2$ come from the parametrization (9). Note that, if ${{{\mathcal {L}}}}$ has $m < n$ distinct eigenvalues, this formula can be adjusted as in the end of the proof of Theorem 2.4, and the requirement after step 1 can be dropped. Later in the algorithm, one replaces *n* with *m*. It is necessary to clear denominators in order to obtain polynomials (step 5). The saturation by the LCD *D* avoids spurious components arising from this step. Finally, $E_3$ accounts for toric relations between the $z_i$ arising from $\mathbb {Q}$-linear relations among the $\lambda _i$. If no such relations exist, then Theorem 3.5 ensures that the assignment $E_3 \leftarrow \emptyset$ in step 9 is correct.

Steps 6 and 7 in Algorithm 1 require a detailed discussion. Further below we shall explain the ${\mathbb {Q}}$-linear independence of eigenvalues, how to check this, and how to compute $E_3$. Ignoring this for now, one can also run Algorithm 1 with $E_3 = \emptyset$. Then step 13 still returns polynomials that vanish on the Gibbs variety $\textrm{GV}({\mathcal {L}})$ but these may cut out a larger variety.

We implemented Algorithm 1 in Julia (v1.8.3), using Oscar.jl [21], and tested it on many examples. The code is available at https://mathrepo.mis.mpg.de/GibbsManifolds.

### Example 3.3

The Gibbs variety $\textrm{GV}({{{\mathcal {L}}}})$ for the LSSM ${{{\mathcal {L}}}}$ in (1) has the parametrization

$$\begin{aligned} \phi= & {} \sum _{i=1}^{3}\dfrac{z_i}{q(\lambda _i, y_1, y_2, y_3)} \left[ \begin{array}{ccc} p_{11}(\lambda _i, y_1, y_2, y_3)&{} p_{12}(\lambda _i, y_1, y_2, y_3) &{} p_{13}(\lambda _i, y_1, y_2, y_3) \\ p_{12}(\lambda _i, y_1, y_2, y_3)&{} p_{22}(\lambda _i, y_1, y_2, y_3) &{} p_{23}(\lambda _i, y_1, y_2, y_3) \\ p_{13}(\lambda _i, y_1, y_2, y_3)&{} p_{23}(\lambda _i, y_1, y_2, y_3) &{} p_{33}(\lambda _i, y_1, y_2, y_3) \\ \end{array}\right] , \end{aligned}$$

where

$$\begin{aligned}{} & {} \begin{array}{llll} &{} q &{}=&{} 2y_1^2 {+} 6y_1y_2 {+} 2y_2^2 {+} 6y_1y_3 {+} 6y_2y_3 + 2y_3^2 - 6y_1\lambda - 6y_2\lambda - 6y_3\lambda + 3\lambda ^2,\\ &{} p_{11} &{}=&{}y_1^2 + 2y_1y_2 + y_2^2 + 2y_1y_3 + 2y_2y_3 - 2y_1\lambda - 2y_2\lambda - 2y_3\lambda + \lambda ^2,\\ &{} p_{12} &{}=&{} -y_1^2 - y_1y_2 - y_1y_3 + y_2y_3 + y_1\lambda ,\\ &{} p_{13} &{}=&{} -y_1y_2 - y_2^2 + y_1y_3 - y_2y_3 + y_2\lambda ,\\ &{} p_{22} &{}=&{} y_1^2 + 2y_1y_2 + 2y_1y_3 + 2y_2y_3 + y_3^2 - 2y_1\lambda - 2y_2\lambda - 2 y_3\lambda + \lambda ^2,\\ &{} p_{23} &{}=&{} y_1 y_2 - y_1y_3 - y_2y_3 - y_3^2 + y_3\lambda ,\\ &{} p_{33} &{}=&{} 2y_1y_2 + y_2^2 + 2y_1y_3 + 2y_2y_3 + y_3^2 - 2y_1\lambda - 2 y_2\lambda - 2y_3\lambda + \lambda ^2. \end{array} \end{aligned}$$

Our Julia code for Algorithm 1 easily finds the cubic polynomial defining $\textrm{GV}(\mathcal {L)}$.$\diamond$

In spite of such successes, symbolic implicitization is limited to small *n* and *d*. Numerical computations can help, in some cases, to find equations for more challenging Gibbs varieties.

### Example 3.4

We consider the LSSM of $4 \times 4$ Hankel matrices with upper left entry zero:

$$\begin{aligned} {{{\mathcal {L}}}} \, = \, \left\{ \begin{bmatrix} 0 &{} y_2 &{} y_3 &{} y_4 \\ y_2 &{} y_3 &{} y_4 &{} y_5\\ y_3 &{} y_4 &{} y_5 &{} y_6\\ y_4 &{} y_5 &{} y_6 &{} y_7 \end{bmatrix} : \, (y_2, \ldots , y_7) \in {\mathbb {R}}^6 \right\} . \end{aligned}$$

Algorithm 1 failed to compute its Gibbs variety. We proceed using numerics as follows. Fix a degree $D > 0$ and let $$N = \left( {\begin{array}{c}9 + D \\ D\end{array}}\right) $$ be the number of monomials in the 10 unknowns $x_{11}, \ldots , x_{44}$. We create $M \ge N$ samples on $\textrm{GM}({{{\mathcal {L}}}})$ by plugging in random values for the six parameters $y_i$ and applying the matrix exponential. Finding all vanishing equations of degree *D* on these samples amounts to computing the kernel of an $M \times N$ Vandermonde matrix. If this matrix has full rank, then there are no relations of degree *D*. We implemented this procedure in Julia. In our example, Theorem 2.4 says that $\textrm{GV}({{{\mathcal {L}}}})$ is contained in a hypersurface. Using our numerical method, we find one defining equation of degree $D = 6$. We used $M = 5205 \ge N = 5005$ samples. Our sextic has 853 terms with integer coefficients:

$$\begin{aligned}{} & {} x_{11}^3 x_{22} x_{24} x_{34}-x_{11}^3 x_{23}^2 x_{34} -x_{11}^3 x_{23} x_{24}^2+x_{11}^3 x_{23} x_{24} x_{33}+ \\{} & {} \quad \cdots + 3 x_{23} x_{24}^2 x_{33} x_{34}^2+x_{24}^4 x_{33} x_{34}-x_{24}^3 x_{33}^2 x_{34}. \end{aligned}$$

Its Newton polytope has the f-vector $(456, 5538, 21,560, 41,172, 44,707, 29,088, 11,236, 2370, 211)$. In fact, the Gibbs variety in this example is precisely the hypersurface defined by this sextiv. This follows from a result of the first author, namely [23, Theorem 2.6], which appeared while the present article was under review.

Note that the package Oscar.jl conveniently allows to perform symbolic and numerical implicitization and polyhedral computations in the same programming environment.

We emphasize that our numerical Julia code is set up to find *exact* integer coefficients. For this, we first normalize the numerical approximation of the coefficient vector by setting its first (numerically) nonzero entry to one. Then we rationalize the coefficients using the built in command rationalize in Julia, with error tolerance tol = 1e-7. Correctness of the result is proved by checking that the resulting polynomial vanishes on the parametrization.$\diamond$

We now turn to $\mathbb {Q}$-linear relations among eigenvalues of ${\mathcal {L}}$. Our arithmetic discussion begins with a version of [1, (SP)], which is well-known in transcendental number theory:

### Theorem 3.5

(Ax-Schanuel) If the eigenvalues $\lambda _1,\ldots ,\lambda _n$ of the LSSM $\,{\mathcal {L}}$ are ${\mathbb {Q}}$-linearly independent, then $e^{\lambda _1}, \ldots , e^{\lambda _n}$ are algebraically independent over the field $\,{\mathbb {C}}(y_1,\ldots ,y_d)$.

In our situation, the eigenvalues $\lambda _1,\ldots ,\lambda _n$ are algebraic over ${\mathbb {C}}(y_1,\ldots ,y_d)$. We can therefore conclude that, under the assumptions of Theorem 3.5, their exponentials $e^{\lambda _1}, \ldots , e^{\lambda _n}$ are algebraically independent over ${\mathbb {C}}(\lambda _1,\ldots ,\lambda _n)$.

On the other hand, suppose that the eigenvalues of ${\mathcal {L}}$ satisfy some non-trivial linear relation over ${\mathbb {Q}}$. We can then find nonnegative integers $\alpha _i$ and $\beta _j$, not all zero, such that

10 $$\begin{aligned} \sum _{i=1}^n \alpha _i \lambda _i \,\,=\,\, \sum _{j=1}^n \beta _j \lambda _j. \end{aligned}$$

This implies that the exponentials of the eigenvalues satisfy the toric relations

11 $$\begin{aligned} \prod _{i=1}^n z_i^{\alpha _i} \,\, = \,\, \prod _{j=1}^n z_j^{\beta _j}. \end{aligned}$$

The linear relations (10) can be found from the ideal $\langle E_1' \rangle$ in step 2 which specifies that the $\lambda _i$ are the eigenvalues of *A*(*y*). This ideal is radical if we assume that ${\mathcal {L}}$ has distinct eigenvalues. We compute the prime decomposition of the ideal over ${\mathbb {Q}}$. All prime components are equivalent under permuting the $\lambda _i$, so we replace $\langle E_1' \rangle$ by any of these prime ideals in step 3. We compute (10) as the linear forms in that prime ideal. Using (11), we compute the toric ideal $\langle E_3 \rangle$ in step 7, which is also prime. This ideal defines a toric variety $W'$, whose $S_n$-orbit is the variety *W* in Remark 3.2. We arrive at the following result.

### Theorem 3.6

Let ${\mathcal {L}} \subset {\mathbb {S}}^n$ be an LSSM with distinct eigenvalues. The Gibbs variety $\textrm{GV}({\mathcal {L}})$ is irreducible and unirational, and the ideal *J* found in Algorithm 1 is its prime ideal.

### Proof

Sylvester’s formula yields a rational parametrization $\psi$ of $\textrm{GV}({\mathcal {L}})$ with parameters $y_1,\ldots ,y_d,z_1,\ldots ,z_n$. The parameters $\lambda _i$ in (9) can be omitted: the entries in the image are multisymmetric in $(\lambda _i,z_i)$, so that they can be expressed in terms of elementary symmetric polynomials of the $\lambda _i$ [2, Theorem 1]. The point $(z_1, \ldots , z_n)$ lies on the toric variety $W'$ defined above. The domain ${\mathbb {C}}^d \times W'$ of $\psi$ is an irreducible variety, and it is also rational. The image of $\psi$ is the Gibbs variety $\textrm{GV}({\mathcal {L}})$, which is therefore unirational and irreducible. The ideals given by $E_1$ and $E_2$ in Algorithm 1 are prime, after saturation, and elimination in step 12 preserves primality. Hence the output in *J* in step 13 is the desired prime ideal. $\square$

We define the *Galois group* $G_{{\mathcal {L}}}$ of an LSSM ${\mathcal {L}}$ to be the Galois group of the characteristic polynomial $P_{\mathcal {L}}(\lambda ,y)$ over the field ${\mathbb {Q}}(y_1,\ldots ,y_d)$. Note that $G_{\mathcal {L}}$ is the subgroup of the symmetric group $S_n$ whose elements are permutations that fix each associated prime of $\langle E_1' \rangle$. Hence the index of the Galois group $G_{\mathcal {L}}$ in $S_n$ is the number of associated primes. In particular, the Galois group equals $S_n$ if and only if the ideal $\langle E_1' \rangle$ formed in step 2 is prime.

The existence of linear relations (10) depends on the Galois group $G_{\mathcal {L}}$. If the Galois group is small then the primes of $\langle E_1 \rangle$ are large, and more likely to contain linear forms. There is a substantial literature in number theory on this topic. See [11, 12] and the references therein. For instance, by Kitaoka [17, Proposition 2], there are no linear relations if *n* is prime, or if $n \ge 6$ and the Galois group is $S_n$ or $A_n$. If this holds, $E_3 = \emptyset$ in step 9 of Algorithm 1.

The computation of Galois groups is a well-studied topic in symbolic computation and number theory. Especially promising are methods based on numerical algebraic geometry (e.g. in [13]). These fit well with the approach to implicitization in Example 3.4. For a future theoretical project, it would be very interesting to classify LSSMs by their Galois groups.

### Remark 3.7

We briefly comment on how to adjust Algorithm 1 to compute the Gibbs variety of an ASSM ${{{\mathcal {L}}}}$ with $A_0 \ne 0$. In this case, algebraic relations between $e^{\lambda _1}, \ldots , e^{\lambda _n}$ come from ${\mathbb {Q}}$-linear relations between the eigenvalues of ${{{\mathcal {L}}}}$, but this time modulo ${\mathbb {C}}$: an affine relation $\sum \alpha _i \lambda _i = \sum \beta _j \lambda _j + \gamma$ gives $z^\alpha - e^\gamma \cdot z^\beta = 0$, where $z_i = e^{\lambda _i}$, $\alpha _i, \beta _j \in {\mathbb {Z}}_{\ge 0}$, $\gamma \in {\mathbb {C}}$. Here $\gamma$ is a ${\mathbb {Q}}$-linear combination of eigenvalues of $A_0$. Theorem 3.6 holds for ASSMs as well, provided that these ${\mathbb {Q}}$-linear relations modulo ${\mathbb {C}}$ can be computed in practice. This can usually not be done over ${\mathbb {Q}}$. We leave this algorithmic challenge for future research.

## Pencils of quadrics

In this section we study the Gibbs variety $\textrm{GV}({\mathcal {L}})$ where ${\mathcal {L}} \subset {\mathbb {S}}^n$ is a pencil of quadrics, i.e. an LSSM of dimension $d=2$. We follow the exposition in [8], where pencils ${\mathcal {L}}$ are classified by Segre symbols. The *Segre symbol* $\,\sigma =\sigma ({\mathcal {L}})$ is a multiset of partitions that sum up to *n*. It is computed as follows: Pick a basis $\{A_1, A_2\}$ of ${\mathcal {L}}$, where $A_2$ is invertible, and find the Jordan canonical form of $A_1 A_2^{-1}$. Each eigenvalue determines a partition, according to the sizes of the corresponding Jordan blocks. The multiset of these partitions is the Segre symbol $\sigma$.

We use the canonical form in [8, Section 2]. Suppose the Segre symbol is $\sigma = [\sigma _1,\ldots ,\sigma _r]$, where the *i*th partition $\sigma _{i}$ equals $(\sigma _{i,1} \ge \sigma _{i,2} \ge \cdots \ge \sigma _{i,n} \ge 0)$. There are *r* groups of blocks, one for each eigenvalue $\alpha _i$ of $A_1 A_2^{-1}$. The *j*th matrix in the *i*th group is the $\sigma _{i,j} \times \sigma _{i,j}$ matrixThere are 13 Segre symbols for $n=4$; see [8, Example 3.1]. It is instructive to compute their Gibbs varieties. All possible dimensions, 2, 3, 4 and 5, are attained. Dimension 2 arises for the diagonal pencil ${\mathcal {L}}_\sigma = \textrm{diag}(\alpha _1 y_1{+}y_2, \alpha _2 y_1 {+} y_2, \alpha _3 y_1 {+} y_2, \alpha _4 y_1{+} y_2)$, with Segre symbol $\sigma = [1,1,1,1]$. When the $\alpha _i$ are distinct integers, $\textrm{GV}({\mathcal {L}}_\sigma ) = \textrm{GM}({\mathcal {L}}_\sigma )$ is a toric surface. This is similar to (2). Dimension 5 arises for $\sigma = [4]$, which was presented in Example 2.3.

The following examples, also computed with Algorithm 1, exhibit the dimensions 5, 4, 3.

### Example 4.1

Consider the Segre symbol $\sigma = [3,1]$. The canonical pencil ${{{\mathcal {L}}}}_{[3,1]}$ is spanned byHere, $\dim \textrm{GV}({{{\mathcal {L}}}}_{[3,1]}) = 5$, the upper bound in Theorem 2.4. Algorithm 1 produces the ideal

$$\begin{aligned} J \, = \,\bigl \langle \, x_{14},x_{24},x_{34},\,x_{13}-x_{22}+x_{33},\,x_{12}^2-x_{11}x_{22}-x_{12}x_{23}+x_{11}x_{33}+x_{22}x_{33}-x_{33}^2 \,\bigr \rangle . \end{aligned}$$

If $\alpha _1= \alpha _2$, then the Segre symbol changes to $\sigma = [(3,1)]$. We now find the additional cubic

12 $$\begin{aligned} x_{11}x_{22}x_{33}+ 2x_{12}x_{13}x_{23} - x_{13}^2 x_{22} - x_{11}x_{23}^2 - x_{12}^2 x_{33} \,-\, x_{44} \quad \in \,\,\, J. \end{aligned}$$

This cuts down the dimension by one, and we now have $\dim \textrm{GV}({{{\mathcal {L}}}}_{[(3,1)]}) = 4$.$\diamond$

### Example 4.2

Consider the Segre symbol $\sigma = [(2,2)]$. The pencil ${\mathcal {L}}_{[(2,2)]}$ is spanned byA version of Algorithm 1 for LSSMs with multiple eigenvalues produces the ideal

$$\begin{aligned} J \, = \, \langle \, x_{11}-x_{33},\,x_{12}-x_{34},\,x_{22} - x_{44},\, x_{13}, \, x_{14},\, x_{23},\,x_{24}\,\rangle . \end{aligned}$$

The Gibbs variety $\textrm{GV}({{{\mathcal {L}}}}_{[(2,2)]})$ is 3-dimensional and equals the Gibbs plane $\textrm{GP}({{{\mathcal {L}}}}_{[(2,2)]})$.$\diamond$

The cubic (12) which distinguishes the Segre symbols [3, 1] and [(3, 1)] is explained by the following result. This applies not just to pencils but to all ASSMs with block structure.

### Proposition 4.3

Let ${{{\mathcal {L}}}}$ be a block-diagonal ASSM with *r* blocks $X_i(y)$ of size $\tau _i$, where $\tau _1 + \cdots + \tau _r = n$. The Gibbs plane $\textrm{GP}({{{\mathcal {L}}}})$ is contained in ${\mathbb {S}}^{\tau _1} \times \cdots \times {\mathbb {S}}^{\tau _r} \subset {\mathbb {S}}^n$. Moreover, with the notation $${{{\mathcal {J}}}} = \{ \{i,j\} \in \left( {\begin{array}{c}[r]\\ 2\end{array}}\right) : \textrm{trace}(X_i(y)) = \textrm{trace}(X_j(y)) \}$$, we have

$$\begin{aligned} \textrm{GV}({{{\mathcal {L}}}}) \, \subseteq \, \{ (X_1, \ldots , X_r) \in \textrm{GP}({{{\mathcal {L}}}}) : \, \det (X_i) = \det (X_j) \text { for all } \,\{i,j \} \in {{{\mathcal {J}}}} \}. \end{aligned}$$

### Proof

Block-diagonal matrices are exponentiated block-wise. The entries outside the diagonal blocks are zero. The statement follows from $\,\textrm{det}(\textrm{exp}(X_i(y))) = \textrm{exp}(\textrm{trace}(X_i(y)))$. $\square$

Proposition 4.3 applies to the canonical pencil ${\mathcal {L}}_\sigma$ of any Segre symbol $\sigma$. First of all, for all indices (*i*, *j*) outside the diagonal blocks, we have $x_{ij} = 0$ on the Gibbs plane $\textrm{GP}({{{\mathcal {L}}}}_\sigma )$. Next, one has equations for the exponential of a single block, like those in Theorem 4.4 below. Finally, there are equations that link the blocks corresponding to entries $\sigma _{ij}$ of the same partition $\sigma _i$. Some of these come from trace equalities between blocks of ${{{\mathcal {L}}}}_\sigma$, and this is the scope of Proposition 4.3. In particular, blocks *ij* and *ik* for which $\sigma _{ij} = \sigma _{ik} \, \textrm{mod} \, 2$ exponentiate to $X_{ij} \in {\mathbb {S}}^{\sigma _{ij}}_+$ and $X_{ik} \in {\mathbb {S}}^{\sigma _{ik}}_+$ with equal determinant. We saw this in (12). In all examples we computed, the three classes of equations above determine the Gibbs variety.

We now derive the equations that hold for the exponential of a single block. To this end, we fix $\sigma = [n]$ with $\alpha _1 = 0$. The canonical LSSM ${{{\mathcal {L}}}}_{[n]}$ consists of the symmetric matricesThe case $n=4$ was featured in Example 2.3. In what follows we generalize that example. By convention, we assume $x_{ij}=0$ if $i > n$ or $j>n$

### Theorem 4.4

The following linear equations hold on the Gibbs plane $\textrm{GP}({\mathcal {L}}_{[n]})$:

13 $$\begin{aligned} x_{i-1,j}+x_{i+1,j}\,\,=\,\, x_{i,j-1}+x_{i,j+1} \quad \hbox {for }\,2\leqslant i < j \leqslant n. \end{aligned}$$

The $2 \times 2$-minors of the following $2\times (n-1)$ matrix vanish on the Gibbs variety $\textrm{GV}({\mathcal {L}}_{[n]})$:

14 $$\begin{aligned} D(X) \,\, = \,\,\, \begin{bmatrix} x_{11} &{} x_{12} &{} x_{22} &{}\ldots &{}\\ x_{12} &{} x_{22} &{} x_{23} &{}\ldots &{}\\ \end{bmatrix} - \begin{bmatrix} x_{n,n} &{} x_{n-1,n} &{} x_{n-1,n-1}&{}\ldots &{}\\ 0 &{} x_{n,n} &{} x_{n-1,n}&{} \ldots &{}\\ \end{bmatrix}. \end{aligned}$$

If the Galois group $$G_{{\mathcal {L}}_{[n]}}$$ is the symmetric group $$ S_n$$, then the prime ideal of $$\textrm{GV}({\mathcal {L}}_{[n]})$$ is generated by (13) and (14), and we have $$\textrm{dim}\,\textrm{GP}({\mathcal {L}}_n) = 2n-1$$ and $$\textrm{dim}\,\textrm{GV}({\mathcal {L}}_{[n]}) = n+1$$.

### Remark 4.5

We conjecture that $G_{{\mathcal {L}}_{[n]}} = S_n$. This was verified computationally for many values of *n*, but we currently do not have a proof that works for all *n*. This gap underscores the need, pointed out at the end of Sect. 3, for a study of the Galois groups of LSSMs.

### Proof

We claim that the linear equations (13) hold for every non-negative integer power of *Y*. This implies that they hold for $\textrm{exp}(Y)$. We will show this by induction. The equations clearly hold for $Y^0 = \textrm{id}_n$. Suppose they hold for $(m_{ij}) = M = Y^{k}$. Write $(b_{ij})=B:= Y^{k+1} = M Y$.

The two-banded structure of *Y* implies $b_{i,j} = y_1\cdot m_{i,n-j+1} + y_2\cdot m_{i,n-j+2}$ for $1 \leqslant i < j$. The following identity holds for $2 \leqslant i < j$, and it shows that $\textrm{exp}(Y)$ satisfies the equations (13):

$$\begin{aligned}{} & {} b_{i-1,j}-b_{i,j-1}-b_{i,j+1}+b_{i+1,j} \,\,=\,\, y_1\cdot m_{i-1,n-j+1} + y_2\cdot m_{i-1,n-j+2} - y_1\cdot m_{i,n-j+2} \\{} & {} \qquad - y_2\cdot m_{i,n-j+3} - y_1\cdot m_{i,n-j} - y_2\cdot m_{i,n-j+1} + y_1\cdot m_{i+1, n-j+1} + y_2\cdot m_{i+1, n-j+2} \\{} & {} \quad =\,\,y_1\cdot (m_{i-1,n-j+1}-m_{i,n-j+2}-m_{i, n-j}+m_{i+1,n-j+1}) \\ {}{} & {} \qquad + y_2\cdot (m_{i-1,n-j+2} - m_{i,n-j+3} - m_{i,n-j+1} + m_{i+1,n-j+2}) \,\,=\,\, 0. \end{aligned}$$

We next consider the matrix *D*(*X*) in (14). We must show that *D*(*X*) has rank $\le 1$ for $X \in \textrm{GV}({\mathcal {L}}_{[n]})$. We claim that the rows of $D(Y^k)$ are proportional with the same coefficient for all $k \in {\mathbb {Z}}_{\ge 0}$. This will imply that the rows of $D(\exp {(Y}))$ are proportional. For the proof, let $\textbf{v}_1$ and $\textbf{v}_2$ be the rows of *D*(*B*), where $B = Y^k$. We will show that ${y_1 \textbf{v}_1 + y_2 \textbf{v}_2 = 0}$.

First note that $D(\textrm{id}_n) = 0$. Also note that each column of *D*(*B*) has the form

$$\begin{aligned} \begin{bmatrix} b_{i,i}-b_{n+1-i,n+1-i}\\ b_{i,i+1} - b_{n+1-i,n+2-i}\\ \end{bmatrix} \quad \textrm{or} \quad \begin{bmatrix} b_{i,i+1} - b_{n-i,n+1-i}\\ b_{i+1,i+1} - b_{n-i+1,n-i+1}\\ \end{bmatrix}. \end{aligned}$$

We start with the left case. We must show $y_1(b_{i,i}-b_{n+1-i,n+1-i}) + y_2(b_{i,i+1} - b_{n+1-i,n+2-i}) = 0$.

Recall from above that $b_{i,j}=y_1\cdot m_{i,n-j+1}+y_2\cdot m_{i,n-j+2}$, where $(m_{i,j}) = M = Y^{k-1}$ for $i < j$. Using this and the fact that the powers of *Y* are symmetric, we write

$$\begin{aligned}{} & {} y_1(b_{i,i}-b_{n+1-i,n+1-i}) + y_2(b_{i,i+1} - b_{n+1-i,n+2-i}) \\{} & {} \quad = y_1((y_1\cdot m_{i,n-i+1} + y_2\cdot m_{i,n-i+2})-(y_1\cdot m_{n+1-i,i} + y_2\cdot m_{n+1-i,i+1})) \\{} & {} \qquad + y_2((y_1\cdot m_{i,n-i} + y_2 \cdot m_{i,n-i+1})-(y_1\cdot m_{n+1-i,i-1}+y_2\cdot m_{n+1-i,i})) \\{} & {} \quad = y_1 y_2(m_{i,n-i+2}-m_{i+1,n+1-i}+m_{i,n-i} -m_{n+1-i,i-1}) = 0, \end{aligned}$$

where the last equality follows from (13). Now, for the second case we have

$$\begin{aligned}{} & {} y_1(b_{i,i+1} - b_{n-i,n+1-i}) + y_2(b_{i+1,i+1} - b_{n-i+1,n-i+1}) \\ {}{} & {} \quad = y_1(y_1\cdot m_{i,n-i} + y_2\cdot m_{i,n-i+1} - y_1\cdot m_{n-i,i} - y_2\cdot m_{n-i,i+1}) \\ {}{} & {} \qquad + y_2(y_1\cdot m_{i+1,n-i} + y_2\cdot m_{i+1,n-i+1}-y_1\cdot m_{n-i+1,i} - y_2\cdot m_{n-i+1,i+1}) = 0. \end{aligned}$$

This proves that the $2 \times 2$ minors of *D*(*X*) vanish on the Gibbs variety $\,\textrm{GV}({\mathcal {L}}_{[n]} )$.

Suppose now that the eigenvalues of *Y* are $\mathbb {Q}$-linearly independent. We can check this directly for $n \le 5$. For $n \ge 6$ it follows from our hypothesis $G_{{\mathcal {L}}_{[n]}} = S_n$, by [17, Proposition 2]. That hypothesis implies $\textrm{dim} \,\textrm{GV}({\mathcal {L}}_{[n]} )= n+1$, by Theorems 2.4 and 3.5.

For the primality statement, we note that the matrix *D*(*X*) is 1-generic in the sense of Eisenbud [6, 7]. By [6, Theorem 1], the $2 \times 2$-minors of *D*(*X*) generate a prime ideal of codimension $n-2$ in the coordinates of the $(2n-1)$-dimensional space given by (13). The equality of dimensions yields $\dim \textrm{GP}({\mathcal {L}}_{[n]}) = 2n-1$, and we conclude that our linear and quadratic constraints generate the prime ideal of $\textrm{GV}({\mathcal {L}}_{[n]})$. $\square$

Theorem 2.4 and its refinement in Remark 3.2 furnish an upper bound on the dimension of any Gibbs variety. This raises the question when this bound is attained. In what follows, we offer a complete answer for $d=2$. Let ${\mathcal {L}}$ be a pencil with eigenvalues $\lambda _i(y)$, and let *W* denote the Zariski closure in $\mathbb {R}^n$ of the set of points $\textrm{exp}(\lambda (y)) = (e^{\lambda _1(y)}, \ldots , e^{\lambda _n(y)})$, $y \in \mathbb {R}^2$.

### Theorem 4.6

Let $${\mathcal {L}} = \textrm{span}_{{\mathbb {R}}}(A_1,A_2)$$, where $$A_1 A_2 \not = A_2 A_1$$. Then $$\textrm{dim} \,\textrm{GV}({\mathcal {L}}) = \textrm{dim}(W)+1$$. In particular, if the Galois group $$G_{{\mathcal {L}}} $$ is the symmetric group $$ S_n$$ then $$\textrm{dim} \,\textrm{GV}({\mathcal {L}}) = n+1$$.

### Proof

We claim that the fibers of the map $\phi : V\times W \rightarrow {\mathbb {S}}^n$ defined by (9) are one-dimensional. Let $B \in \phi (V \times W)$ and consider any point $p=(y_1,y_2,\lambda _1,\ldots ,\lambda _n,z_1,\ldots ,z_n) \in \phi ^{-1}(B)$. The condition that *p* lies in the fiber $\phi ^{-1}(B)$ is equivalent to

1. $z_1,\ldots , z_n$ are the eigenvalues of *B*, and
2. $X = y_1A_1+y_2A_2$ and *B* have the same eigenvectors, and
3. $\lambda _1, \ldots , \lambda _n$ are the eigenvalues of *X*.

Condition (1) follows from Theorem 3.1 for $f = \textrm{exp}$. It implies that there are only finitely many possibilities for the *z*-coordinates of the point *p* in the fiber: up to permutations, they are the eigenvalues of *B*. Condition (3) follows from $(y_1,y_2,\lambda _1,\ldots ,\lambda _n)\in V$. It says that the $\lambda$-coordinates are determined, up to permutation, by $y_1,y_2$. Therefore, it suffices to show that the matrices in ${{{\mathcal {L}}}}$ whose eigenvectors are those of *B* form a one-dimensional subvariety.

Symmetric matrices have common eigenvectors if and only if they commute. Define $S = \{ X = y_1 A_1 + y_2 A_2 \in {{{\mathcal {L}}}} \,: X \cdot B = B \cdot X \} \subset {{{\mathcal {L}}}}$. This is a pairwise commuting linear subspace. Note that *S* contains a nonzero matrix *X*, since there is a point in $\phi ^{-1}(B)$ whose *y*-coordinates define a nonzero matrix in ${{{\mathcal {L}}}}$. Therefore $\dim S \ge 1$. Since $A_1A_2 \ne A_2A_1$, we also have $\dim S \le 1$. Hence $\dim S = \dim \phi ^{-1}(B) = 1$. Moreover, the upper bound $\dim W + 1$ for the dimension of $\textrm{GV}({{{\mathcal {L}}}})$, which is given by Remark 3.2, is attained in our situation. $\square$

## Convex optimization

In this section we show how Gibbs manifolds arise from entropic regularization in optimization (cf. [26]). We fix an arbitrary linear map $\pi : {\mathbb {S}}^n \rightarrow \mathbb {R}^d$. This can be written in the form

$$\begin{aligned} \pi (X) = \bigl ( \langle A_1, X \rangle , \langle A_2, X \rangle , \ldots , \langle A_d, X \rangle \bigr ). \end{aligned}$$

Here the $A_i \in {\mathbb {S}}^n$, and $\langle A_i, X \rangle := \textrm{trace}(A_i X)$. The image $\pi ({\mathbb {S}}^n_+)$ of the PSD cone ${\mathbb {S}}^n_+$ under our linear map $\pi$ is a *spectrahedral shadow*. Here it is a full-dimensional semialgebraic convex cone in $\mathbb {R}^d$. Interestingly, $\pi ({\mathbb {S}}^n_+)$ can fail to be closed, as explained in [16].

*Semidefinite programming (SDP)* is the following convex optimization problem:

15 $$\begin{aligned} \textrm{Minimize} \quad \langle C, X \rangle \quad \hbox {subject to} \quad X \in {\mathbb {S}}^n_+ \,\text { and }\, \pi (X) = b. \end{aligned}$$

See e.g. [19, Chapter 12]. The instance (15) is specified by the cost matrix $C \in {\mathbb {S}}^n$ and the right hand side vector $b \in \mathbb {R}^d$. The feasible region ${\mathbb {S}}^n_+ \cap \pi ^{-1}(b)$ is a *spectrahedron*. The SDP problem (15) is feasible, i.e. the spectrahedron is non-empty, if and only if $\,b$ is in $\pi ({\mathbb {S}}^n_+)$.

Consider the LSSM ${\mathcal {L}} = \textrm{span}_\mathbb {R}(A_1, \ldots , A_d)$. We usually assume that ${\mathcal {L}}$ contains a positive definite matrix. This hypothesis ensures that each spectrahedron $\pi ^{-1}(b)$ is compact.

As a natural extension of [26, eqn (2)], we now define the entropic regularization of SDP:

16 $$\begin{aligned} \textrm{Minimize} \quad \langle C, X \rangle \,-\, \epsilon \cdot h(X) \quad \hbox {subject to} \quad X \in {\mathbb {S}}^n_+ \,\text { and } \,\pi (X) = b. \end{aligned}$$

Here $\epsilon > 0$ is a small parameter, and *h* denotes the *von Neumann entropy*, here defined as

$$\begin{aligned} h: \, {\mathbb {S}}^n_+ \,\rightarrow \, \mathbb {R}, \,\, X \,\mapsto \, \textrm{trace} \bigl ( X - X \cdot \textrm{log}(X) \bigr ). \end{aligned}$$

We note that *h* is invariant under the action of the orthogonal group on ${\mathbb {S}}^n_+$. This implies that $h(X) = \sum _{i=1}^n (\lambda _i-\lambda _i \textrm{log}(\lambda _i) )$, where $\lambda _1,\ldots ,\lambda _n$ are the eigenvalues of *X*. Hence the von Neumann entropy *h* is the matrix version of the entropy function on $\mathbb {R}^n_+$ used in [26].

Our next result makes the role of Gibbs manifolds in semidefinite programming explicit. The following ASSM is obtained by incorporating $\epsilon$ and the cost matrix *C* into the LSSM:

$$\begin{aligned} {\mathcal {L}}_\epsilon \,:= \,\, {\mathcal {L}}- \frac{1}{\epsilon } C \quad \hbox {for any} \,\,\epsilon > 0. \end{aligned}$$

Here we allow the case $\epsilon = \infty$, where the dependency on *C* disappears and the ASSM is simply the LSSM, i.e. ${\mathcal {L}}_\infty = {\mathcal {L}}$. The following theorem is the main result in this section.

### Theorem 5.1

For $b \in \pi ({\mathbb {S}}^n_+)$, the intersection of $\pi ^{-1}(b)$ with the Gibbs manifold $\textrm{GM}({\mathcal {L}}_\epsilon )$ consists of a single point $X_\epsilon ^*$. This point is the optimal solution to the regularized SDP (16). For $\epsilon = \infty$, it is the unique maximizer of von Neumann entropy on the spectrahedron $\pi ^{-1}(b)$.

The importance of this result for semidefinite programming lies in taking the limit as $\epsilon$ tends to zero. This limit $\,\textrm{lim}_{\epsilon \rightarrow 0} \,X^*_\epsilon \,$ exists and it is an optimal solution to (15). The optimal solution is unique for generic *C*. Entropic regularization is about approximating that limit.

### Remark 5.2

Theorem 5.1 implies that adding the condition $X \in \textrm{GV}({{{\mathcal {L}}}}_\epsilon )$ to (16) leaves the optimizer unchanged. Hence, if we know equations for the Gibbs variety, we may shrink the feasible region by adding polynomial constraints. Most practical are the affine-linear equations: imposing $X \in \textrm{GP}({{{\mathcal {L}}}}_\epsilon )$ allows to solve (16) on a spectrahedron of lower dimension.

To prove Theorem 5.1, we derive two key properties of the von Neumann entropy:

### Proposition 5.3

The function *h* satisfies:

- *h* is strictly concave on the PSD cone ${\mathbb {S}}^n_+$, and
- the gradient of *h* is the negative matrix logarithm: $\,\nabla (h)(X) = -\textrm{log}(X)$.

### Proof

For (a), we use a classical result by Davis [4]. The function *h* is invariant in the sense that its value *h*(*X*) depends on the eigenvalues of *X*. In fact, it is a symmetric function of the *n* eigenvalues $\lambda _1,\lambda _2,\ldots ,\lambda _n$. This function equals $h(\lambda _1,\lambda _2,\ldots ,\lambda _n) = \sum _{i=1}^n (\lambda _i-\lambda _i \textrm{log}(\lambda _i))$, and this is a concave function $\mathbb {R}^n_+ \rightarrow \mathbb {R}$. The assertion hence follows from the theorem in [4].

For (b) we prove a more general result. For convenience, we change variables $Y = X - \textrm{id}_n$ so that $f(Y) = h(Y + \textrm{id}_n)$ is analytic at $Y = 0$. Fix any function $f: \mathbb {R}\rightarrow \mathbb {R}$ that is analytic in a neighborhood of the origin. Then $Y \mapsto \textrm{trace}(f(Y))$ is a well-defined real-valued analytic function of $n \times n$ matrices $Y = (y_{ij})$ that are close to zero. The gradient of this function is the $n \times n$ matrix whose entries are the partial derivatives $\partial \textrm{trace}( f(Y))/\partial y_{ij}$. We claim that

17 $$\begin{aligned} \nabla \textrm{trace}(f(Y)) \, = \, f'( Y^\top ). \end{aligned}$$

Both sides are linear in *f*, and *f* is analytic, so it suffices to prove this for monomials, i.e.

18 $$\begin{aligned} \nabla \textrm{trace}(Y^k) \, = \, k \cdot (Y^\top )^{k-1} \qquad \hbox {for all integers} \,\, k \ge 1. \end{aligned}$$

Note that $\textrm{trace}(Y^k)$ is a homogeneous polynomial of degree *k* in the matrix entries $y_{ij}$, namely it is the sum over all products $y_{i_1 i_2} y_{i_2 i_3} \cdots y_{i_{k-2} i_{k-1}} y_{i_{k-1} i_1}$ that represent closed walks in the complete graph on *k* nodes. When taking the derivative $\partial /\partial y_{ij}$ of that sum, we obtain *k* times the sum over all walks that start at node *j* and end at node *i*. Here each walk occurs with the factor *k* because $y_{ij}$ can be inserted in *k* different ways to create one of the closed walks above. This polynomial of degree $k-1$ is the entry of the matrix power $Y^{k-1}$ in row *j* and column *i*, so it is the entry of its transpose $(Y^\top )^{k-1}$ in row *i* and column *j*. To prove the proposition, we now apply (17) to the function $f(y) =(y+1) - (y+1) \cdot \textrm{log}(y+1)$. $\square$

If ${\mathcal {L}}={\mathcal {D}}$ consists of diagonal matrices then the Gibbs manifold $\textrm{GM}({\mathcal {D}})$ is a discrete exponential family [27, §6.2], and $\pi (\textrm{GM}({\mathcal {D}}))$ is the associated convex polytope. This uses the moment map from toric geometry [19, Theorem 8.24]. In particular, if the linear space ${\mathcal {D}}$ is defined over ${\mathbb {Q}}$ then the polytope is rational and the Zariski closure of $\textrm{GM}({\mathcal {D}})$ is the toric variety of that polytope. If the space ${\mathcal {D}}$ is not defined over ${\mathbb {Q}}$ then $\textrm{GM}({\mathcal {D}})$ is an analytic toric manifold, whose Zariski closure is the larger toric variety $\textrm{GV}({\mathcal {D}}) = \textrm{GM}({\mathcal {D}}_{\mathbb {Q}})$ seen in (7).

The key step to proving Theorem 5.1 is a non-abelian version of the toric moment map.

### Theorem 5.4

The restriction of the linear map $\pi : {\mathbb {S}}^n_+ \rightarrow \mathbb {R}^d$ to the Gibbs manifold $\textrm{GM}({\mathcal {L}})$ defines a bijection between $\,\textrm{GM}({\mathcal {L}})$ and the open spectrahedral shadow $\,\textrm{int}( \pi ({\mathbb {S}}^n_+))\,$ in $\,\mathbb {R}^d$.

### Proof

Fix an arbitrary positive definite matrix $X \in \textrm{int}({\mathbb {S}}^n_+)$ and set $b = \pi (X)$. We must show that the spectrahedron $\pi ^{-1}(b)$ contains precisely one point that lies in $\textrm{GM}({\mathcal {L}})$.

Consider the restriction of the von Neumann entropy *h* to the spectrahedron $\pi ^{-1}(b)$. This restriction is strictly concave on the convex body $\pi ^{-1}(b)$ by Proposition 5.3. Therefore *h* attains a unique maximum $X^*$ in the relative interior of $\pi ^{-1}(b)$. The first order condition at this maximum tells us that $\nabla (h)(X^*) = -\textrm{log}(X^*)$ lies in ${\mathcal {L}}$, which is the span of the gradients of the constraints $\langle A_i,X \rangle = b_i$. Hence, the optimal matrix $X^*$ lies in the Gibbs manifold

$$\begin{aligned} \textrm{GM}({\mathcal {L}}) \,\,=\,\, \bigl \{ X \in {\mathbb {S}}^n_+: \textrm{log}(X) \in {\mathcal {L}} \bigr \}. \end{aligned}$$

The assignment $b \mapsto X^*= X^*(b)$ is well defined and continuous on the interior of the cone $\pi ({\mathbb {S}}^n_+)$. We have shown that it is a section of the linear map $\pi$, which means $\pi ( X^*(b)) = b$. It is also surjective onto $\textrm{GM}({{{\mathcal {L}}}})$, because $X^*(\pi (X)) = X$, for $X \in \textrm{GM}({{{\mathcal {L}}}})$. We conclude that $\pi$ defines a homeomorphism between $\textrm{GM}({\mathcal {L}})$ and $\,\textrm{int}( \pi ({\mathbb {S}}^n_+))$. $\square$

### Proof of Theorem 5.1

For any fixed $\epsilon > 0$, the minimizer $X^* = X^*_\epsilon$ of the regularized problem (16) lies in the interior of the spectrahedron $\pi ^{-1}(b)$. This is because the gradient of the entropy function diverges at the boundary (Proposition 5.3). By the same convexity argument as in the proof of Theorem 5.4, the objective function in (16) has only one critical point $X^*$ in the spectrahedron $\pi ^{-1}(b)$. It satisfies the first order optimality conditions, which impose $\,C + \epsilon \cdot \textrm{log}(X^*) \in {\mathcal {L}}$. Therefore $X^* \in \textrm{GM}({\mathcal {L}}_\epsilon )$, and $\,\pi ^{-1}(b) \cap \textrm{GM} ({\mathcal {L}}_\epsilon ) \, = \,\{X^*_\epsilon \}$. $\square$

We can now turn the discussion around and offer a definition of Gibbs manifolds and Gibbs varieties purely in terms of convex optimization. Fix any LSSM ${\mathcal {L}}$ of dimension *d* in ${\mathbb {S}}^n$. This defines a canonical linear map $\pi : {\mathbb {S}}^n_+ \rightarrow {\mathbb {S}}^n/{\mathcal {L}}^\perp \simeq \mathbb {R}^d$. Each fiber $\pi ^{-1}(b)$ is a spectrahedron. If this is non-empty then the entropy *h*(*X*) has a unique maximizer $X^*(b)$ in $\pi ^{-1}(b)$. The Gibbs manifold $\textrm{GM}({\mathcal {L}})$ is the set of these entropy maximizers $X^*(b)$ for $b \in \mathbb {R}^d$. The Gibbs variety $\textrm{GV}({\mathcal {L}})$ is defined by all polynomial constraints satisfied by these $X^*(b)$.

This extends naturally to any ASSM $A_0 + {\mathcal {L}}$. Here we maximize the concave function $h(X) + \langle A_0, X\rangle$ over the spectrahedra $\pi ^{-1}(b)$. The Gibbs manifold $\textrm{GM}(A_0+{\mathcal {L}})$ collects all maximizers, and the Gibbs variety $\textrm{GV}(A_0 + {\mathcal {L}})$ is defined by their polynomial constraints.

### Example 5.5

Let ${\mathcal {L}}$ denote the space of all Hankel matrices $[y_{i+j-1}]_{1 \le i,j \le n}$ in ${\mathbb {S}}^n$. This LSSM has dimension $d = 2n-1$. The linear map $\pi : {\mathbb {S}}^n_+ \rightarrow \mathbb {R}^d$ takes any positive definite matrix *X* to a nonnegative polynomial $b = b(t)$ in one variable *t* of degree $2n-2$. We have $b(t) = (1,t,\ldots ,t^{n-1}) X (1,t,\ldots ,t^{n-1})^\top$, so the matrix *X* gives a sum-of-squares (SOS) representation of *b*(*t*). The fiber $\pi ^{-1}(b)$ is the *Gram spectrahedron* [24] of the polynomial *b*. The entropy maximizer $X^*(b)$ in the Gram spectrahedron is a favorite SOS representation of *b*. The Gibbs manifold $\textrm{GM}({\mathcal {L}})$ gathers the favorite SOS representations for all non-negative polynomials *b*. The Gibbs variety $\textrm{GV}({\mathcal {L}})$, which has dimension $\le 3n-2$, is the tightest outer approximation of $\textrm{GM}({\mathcal {L}})$ that is definable by polynomials in the matrix entries.

In Example 3.4 we saw a variant of ${\mathcal {L}}$, namely the sub-LSSM where the upper left entry of the Hankel matrix was fixed to be zero. If $C =-E_{11}$ is the corresponding negated matrix unit, then (15) is the problem of minimizing *b*(*t*) over $t \in \mathbb {R}$. See [19, Section 12.3] for a first introduction to polynomial optimization via SOS representations. It would be interesting to explore the potential of the entropic regularization (16) for polynomial optimization.$\diamond$

One of the topics of [26] was a scaling algorithm for solving the optimization problem (16) for linear programming (LP), i.e. the case when $A_1,\ldots ,A_d$ are diagonal matrices. This algorithm extends the Darroch-Ratcliff algorithm for Iterative Proportional Fitting in statistics. Combining this with a method for driving $\epsilon$ to zero leads to a numerical algorithm for large-scale LP problems, such as the optimal transport problems in [26, Section 3].

We are hopeful that the scaling algorithm can be extended to the problem (16) in full generality. By combining this with a method for driving $\epsilon$ to zero, one obtains a numerical framework for solving SDP problems such as quantum optimal transport in Sect. 6.

One important geometric object for SDP is the limiting Gibbs manifold, $\textrm{lim}_{\epsilon \rightarrow 0} \,\textrm{GM}({\mathcal {L}}_\epsilon )$. This is the set of optimal solutions, as *b* ranges over $\mathbb {R}^d$. In the case of LP, with *C* generic, it is the simplicial complex which forms the regular triangulation given by *C*. This reveals the combinatorial essence of entropic regularization of LP, as explained in [26, Theorem 7]. From the perspective of *positive geometry*, it would be worthwhile to study $\textrm{lim}_{\epsilon \rightarrow 0}\,\textrm{GM}({\mathcal {L}}_\epsilon )$ for SDP. This set is semialgebraic, and it defines a nonlinear subdivision of the spectrahedral shadow $\pi ({\mathbb {S}}^n_+)$. If we vary the cost matrix *C*, the theory of *fiber bodies* in [18] becomes relevant.

## Quantum optimal transport

In this section we examine a semidefinite programming analogue of the classical optimal transport problem, known as *quantum optimal transport* (QOT). We follow the presentation by Cole, Eckstein, Friedland, and Zyczkowski in [3]. Our notation for the dimensions is as in [26, Section 3.1]. We consider the space ${\mathbb {S}}^{d_1 d_2}$ of real symmetric matrices *X* of size $d_1 d_2 \times d_1 d_2$. Rows and columns are indexed by $[d_1] \times [d_2]$. Thus, we write $X = (x_{ijkl})$, where (*i*, *j*) and (*k*, *l*) are in $[d_1] \times [d_2]$. The matrix being symmetric means that $x_{ijkl} = x_{klij}$ for all indices. Each such matrix is mapped to a pair of two *partial traces* by the following linear map:

$$\begin{aligned} {\mathbb {S}}^{d_1 d_2} \,\rightarrow \, {\mathbb {S}}^{d_1} \times {\mathbb {S}}^{d_2}, \,\,\, X \mapsto (Y,Z), \end{aligned}$$

where the $d_1 {\times } d_1$ matrix $Y = (y_{ik})$ satisfies $y_{ik} = \sum _{j=1}^{d_2} x_{ijkj}$, and the $d_2 {\times } d_2$ matrix $Z = (z_{jl})$ satisfies $z_{jl} = \sum _{i=1}^{d_1} x_{ijil}$. If *X* is positive semidefinite then so are its partial traces *Y* and *Z*. Hence our *marginalization map* restricts to a linear projection of closed convex cones, denoted

19 $$\begin{aligned} \mu :\, {\mathbb {S}}_+^{d_1 d_2} \,\rightarrow \, {\mathbb {S}}_+^{d_1} \times {\mathbb {S}}_+^{d_2}, \,\,\, X \mapsto (Y,Z). \end{aligned}$$

Diagonal matrices in ${\mathbb {S}}_+^{d_1 d_2}$ can be identified with rectangular matrices of format $d_1 \times d_2$ whose entries are nonnegative. The map $\mu$ takes such a rectangular matrix to its row sums and column sums. Hence the restriction of $\mu$ to diagonal matrices in ${\mathbb {S}}_+^{d_1 d_2}$ is precisely the linear map that defines classical optimal transport in the discrete setting of [26, Section 3.1].

The quantum optimal transportation problem (QOT) is the task of minimizing a linear function $X \mapsto \langle C, X \rangle$ over any transportation spectrahedron $\mu ^{-1}(Y,Z)$. This is an SDP. Our main theorem in this section states that the Gibbs manifold of $\mu$ is semialgebraic.

### Theorem 6.1

The Gibbs manifold $\textrm{GM}({\mathcal {L}})$ for QOT is a semialgebraic subset of ${\mathbb {S}}^{d_1d_2}_+$. It consists of all symmetric matrices $Y \otimes Z$, where $Y \in {\mathbb {S}}^{d_1}_+$ and $Z \in {\mathbb {S}}^{d_2}_+$. The Gibbs variety $\textrm{GV}({{{\mathcal {L}}}}) \subset {\mathbb {S}}^{d_1d_2}$ is linearly isomorphic to the cone over the Segre variety $$\,\mathbb {P}^{\left( {\begin{array}{c}d_1+1\\ 2\end{array}}\right) -1} \times \mathbb {P}^{\left( {\begin{array}{c}d_2+1\\ 2\end{array}}\right) -1} $$.

The image of the marginalization map $\mu$ generalizes the polytope $\Delta _{d_1-1} \times \Delta _{d_2-1}$, and the fibers of $\mu$ are quantum versions of transportation polytopes. These shapes are now nonlinear.

### Lemma 6.2

The image of the map $\mu$ is a convex cone of dimension $$\left( {\begin{array}{c}d_1+1\\ 2\end{array}}\right) + \left( {\begin{array}{c}d_2+1\\ 2\end{array}}\right) - 1$$:

20 $$\begin{aligned} \textrm{image}(\mu ) \,\,\, = \,\,\, \bigl \{ (Y,Z) \in {\mathbb {S}}_+^{d_1} \times {\mathbb {S}}_+^{d_2}: \, \textrm{trace}(Y) = \textrm{trace}(Z) \bigr \}. \end{aligned}$$

For any point (*Y*, *Z*) in the relative interior of this cone, the *transportation spectrahedron* $\mu ^{-1}(Y,Z)$ is a compact convex body of dimension $\frac{1}{2} (d_1-1)(d_2-1) (d_1 d_2 + d_1 + d_2 + 2)$.

### Proof of Lemma 6.2

The partial trace map $\mu$ in (19) restricts to tensor products as follows:

21 $$\begin{aligned} \mu (Y \otimes Z) \, = \, \bigl (\, \textrm{trace}(Z) \cdot Y,\, \textrm{trace}(Y) \cdot Z \,\bigr ). \end{aligned}$$

Hence, if $Y \in {\mathbb {S}}^{d_1}_+$ and $Z \in {\mathbb {S}}^{d_2}_+$ satisfy $t = \textrm{trace}(Y) = \textrm{trace}(Z)$ then $\frac{1}{t} Y \otimes Z$ is a positive semidefinite matrix in the fiber $\mu ^{-1}(Y,Z)$. This shows that the image is as claimed on the right hand side of (20). The image is a spectrahedral cone of dimension $$\left( {\begin{array}{c}d_1+1\\ 2\end{array}}\right) {+} \left( {\begin{array}{c}d_2+1\\ 2\end{array}}\right) {-} 1$$. Subtracting this from $$\textrm{dim}\,{\mathbb {S}}^{d_1 d_2}_+ = \left( {\begin{array}{c}d_1 d_2 + 1\\ 2\end{array}}\right) $$ yields the dimension of the interior fibers. $\square$

### Example 6.3

($d_1{=}d_2{=}2$) The map $\mu$ projects positive semidefinite $4 \times 4$ symmetric matrices

$$\begin{aligned} X \,=\, \begin{bmatrix} x_{1111} &{} x_{1112} &{} x_{1121} &{} x_{1122} \\ x_{1112} &{} x_{1212} &{} x_{1221} &{} x_{1222} \\ x_{1121} &{} x_{1221} &{} x_{2121} &{} x_{2122} \\ x_{1122} &{} x_{1222} &{} x_{2122} &{} x_{2222} \end{bmatrix} \end{aligned}$$

onto a 5-dimensional convex cone, given by the direct product of two disks. The formula is

$$\begin{aligned} Y \,=\,\begin{bmatrix} x_{1111}+x_{1212} &{} x_{1121}+x_{1222} \\ x_{1121}+x_{1222} &{} x_{2121}+x_{2222} \end{bmatrix} \quad \textrm{and} \quad Z \,= \, \begin{bmatrix} x_{1111}+x_{2121} &{} x_{1112}+x_{2122} \\ x_{1112}+x_{2122} &{} x_{1212}+x_{2222} \end{bmatrix}. \end{aligned}$$

The fibers of this map $\mu$ are the 5-dimensional transportation spectrahedra $\mu ^{-1}(Y,Z)$.

To illustrate the QOT problem, we fix the margins and the cost matrix as follows:

22 $$\begin{aligned} Y \, = \, \begin{bmatrix} 5 &{} 1 \\ 1 &{} 6 \end{bmatrix} \quad \textrm{and} \quad Z \, = \, \begin{bmatrix} 7 &{} 2 \\ 2 &{} 4 \end{bmatrix} \quad \textrm{and} \quad C \,\, = \,\, y \begin{bmatrix} 2 &{} 3 &{} 5 &{} 7 \\ 3 &{} 11 &{} 13 &{} 17 \\ 5 &{} 13 &{} 23 &{} 29 \\ 7 &{} 17 &{} 29 &{}31 \end{bmatrix}. \end{aligned}$$

We wish to minimize $\langle C, X \rangle$ subject to $\mu (X) = (Y,Z)$. The optimal solution $X^*$ is equal to

$$\begin{aligned}{} & {} \left[ \begin{matrix} 3.579128995196972555885181314 &{}2.148103387337332721011731020 \\ 2.148103387337332721011731020 &{} 1.420871004803027444114818686 \\ 2.671254991031789281229265149 &{} 1.169783821392767632002405371 \\ -2.07566204542024789990696017 &{} -1.671254991031789281229265149 \\ \end{matrix}\right. \\{} & {} \qquad \left. \begin{matrix} 2.671254991031789281229265149 &{} -2.07566204542024789990696017 \\ 1.16978382139276763200240537 &{} \!\!\! -1.671254991031789281229265149 \\ 3.420871004803027444114818686 &{} -0.14810338733733272101173102 \\ -0.14810338733733272101173102 &{} 2.579128995196972555885181314 \\ \end{matrix}\right] . \end{aligned}$$

This matrix has rank 2. The optimal value equals $v = 156.964485798827271035367539305\ldots$. This is an algebraic number of degree 12. Its exact representation is the minimal polynomial

$$\begin{aligned}{} & {} 125 v^{12}-465480 v^{11}+770321646 v^{10}{-}744236670798 v^9 {+}463560077206539 v^8\\{} & {} \quad {-}193865445786866004v^7 +54901023652716544539v^6\\{} & {} \quad - 10330064181552258647604 v^5+1219620644420527588643307 v^4 \\{} & {} \quad - 77994100149206862070472310 v^3+1395374211380010273312826701 v^2 \\{} & {} \quad + 83502957914204004050312708316 v\\{} & {} \quad -2047417613706778627978564647804 \,\,=\,\,0. \end{aligned}$$

This was derived from the KKT equations in [20, Theorem 3]. We conclude that the algebraic degree of QOT for $d_1 = d_2 = 2$ is equal to 12. This is smaller than the algebraic degree of semidefinite programming, which is 42. That is the entry for $m{=}5$ and $n{=}4$ in [20, Table 2].

This drop arises because QOT is a very special SDP. The LSSM for our QOT problem is

23 $$\begin{aligned} {\mathcal {L}} \,\, = \,\, \left\{ \begin{bmatrix} y_1 + y_3 &{} y_5 &{} y_4 &{} 0 \\ y_5 &{} y_1 &{} 0 &{} y_4 \\ y_4 &{} 0 &{} y_2+y_3 &{} y_5 \,\\ 0 &{} y_4 &{} y_5 &{} y_2 \, \end{bmatrix}:\,\, y_1,y_2,y_3,y_4,y_5 \in \mathbb {R}\, \right\} . \end{aligned}$$

This defines our 5-dimensional Gibbs manifold $\textrm{GM}({\mathcal {L}})$ in the 10-dimensional cone ${\mathbb {S}}^4_+$. Theorem 6.1 states that it equals the positive part of the Gibbs variety, i.e. $\textrm{GM}({\mathcal {L}}) = \textrm{GV}({\mathcal {L}}) \cap {\mathbb {S}}^4_+$.

We compute the entropy maximizer inside the 5-dimensional transportation spectrahedron $\mu ^{-1}(Y,Z)$ for the marginal matrices *Y* and *Z* in (22). Notably, its entries are rational:

$$\begin{aligned} \mu ^{-1}(Y,Z) \,\cap \, \textrm{GV}({\mathcal {L}}) \quad = \quad \mu ^{-1}(Y,Z) \,\cap \, \textrm{GM}({\mathcal {L}}) \quad = \quad \left\{ \,\frac{1}{11} \begin{bmatrix} 35 &{} 10 &{} 7 &{} 2 \\ 10 &{} 20 &{} 2 &{} 4 \\ 7 &{} 2 &{} 42 &{} 12 \\ 2 &{} 4 &{} 12 &{} 24 \end{bmatrix}\, \right\} . \end{aligned}$$

### Proof of Theorem 6.1

By linear extension, the equation (21) serves as a definition of the marginalization map $\mu$ on ${\mathbb {S}}^{d_1 d_2}$. We observe the following for the trace inner product on ${\mathbb {S}}^{d_1 d_2}$:

$$\begin{aligned} \begin{array}{ccc} &{} \textrm{trace} \bigl ( (A \otimes \textrm{id}_{d_2}) (Y \otimes Z) \bigr ) \,= \, \textrm{trace}(Z) \cdot \textrm{trace}(AY) &{} \hbox {for all}\,\,\, A \in {\mathbb {S}}^{d_1} \\ \textrm{and} &{} \textrm{trace} \bigl ( (\textrm{id}_{d_1} \otimes B) (Y \otimes Z) \bigr ) \,= \, \textrm{trace}(Y) \cdot \textrm{trace}(BZ) &{} \hbox {for all}\,\,\, B \in {\mathbb {S}}^{d_2}. \\ \end{array} \end{aligned}$$

Therefore, the (*i*, *j*) entry of $\textrm{trace}(Z) \cdot Y$ is obtained as $\frac{1}{2}\langle (E_{ij} + E_{ji}) \otimes \textrm{id}_{d_2}, Y \otimes Z \rangle$, where $E_{ij}$ is the (*i*, *j*)-th matrix unit. A similar observation holds for the entries of $\textrm{trace}(Y) \cdot Z$. This means that $\mu (X)$ is computed by evaluating $\textrm{trace} \bigl ( (A \otimes \textrm{id}_{d_2}) X \bigr )$ and $\textrm{trace} \bigl ( (\textrm{id}_{d_1} \otimes B)X \bigr )$, where *A* ranges over a basis of ${\mathbb {S}}^{d_1}$ and *B* ranges over a basis of ${\mathbb {S}}^{d_2}$. Therefore, we have

24 $$\begin{aligned} {\mathcal {L}} \,\, = \,\, \bigl \{ \,A \otimes \textrm{id}_{d_2}\, + \, \textrm{id}_{d_1} \otimes B:\, A \in {\mathbb {S}}^{d_1}\,\,\textrm{and}\,\, B \in {\mathbb {S}}^{d_2} \,\bigr \}. \end{aligned}$$

Now, the key step in the proof consists of the following formula for the matrix logarithm

$$\begin{aligned} \textrm{log}(Y \otimes Z) \,= \, \textrm{log}(Y) \otimes \textrm{id}_{d_2} \, + \, \textrm{id}_{d_1} \otimes \textrm{log}(Z). \end{aligned}$$

This holds for positive semidefinite matrices *Y* and *Z*, and it is verified by diagonalizing these matrices. By setting $Y = \textrm{exp}(A)$ and $Z = \textrm{exp}(B)$, we now conclude that the Gibbs manifold $\textrm{GM}({\mathcal {L}})$ consists of all tensor products $Y \otimes Z$ where $Y \in {\mathbb {S}}^{d_1}_+$ and $Z \in {\mathbb {S}}^{d_2}_+$.

We have shown that $\textrm{GM}({\mathcal {L}})$ is the intersection of a variety with ${\mathbb {S}}^{d_1d_2}_+$. This variety must be the Gibbs variety $\textrm{GV}({\mathcal {L}})$. More precisely, $\textrm{GV}({\mathcal {L}})$ consists of all tensor products $Y \otimes Z$ where *Y*, *Z* are complex symmetric. This is the cone over the Segre variety, which is the projective variety in $$ \mathbb {P}^{\left( {\begin{array}{c}d_1d_2+1\\ 2\end{array}}\right) -1}$$ whose points are the tensor products ${X = Y \otimes Z}$. $\square$

We have the following immediate consequence of the proof of Theorem 6.1. The entropy maximizers have rational entries. This explains the matrix at the end of Example 6.3

### Corollary 6.4

The Gibbs point for QOT is given by $\frac{Y \otimes Z}{\textrm{trace}(Y)}$, with *Y*, *Z* the given margins.

At this point, it pays off to revisit Sect. 3 and to study its thread for the LSSM in (24).

### Example 6.5

We apply Algorithm 1 to the LSSM ${\mathcal {L}}$ in (23). The eigenvalues of ${\mathcal {L}}$ are distinct, and the ideal $\langle E_1' \rangle$ in step 2 is the intersection of six prime ideals. One of them is

$$\begin{aligned}{} & {} \langle \, \lambda _1+\lambda _2-y_1-y_2-y_3,\lambda _3+\lambda _4-y_1-y_2-y_3,\\{} & {} \quad 2 \lambda _2 \lambda _4-\lambda _2 y_1-\lambda _4 y_1-\lambda _2 y_2-\lambda _4 y_2+2 y_1 y_2\\{} & {} \quad -\lambda _2 y_3-\lambda _4 y_3+y_1 y_3+y_2 y_3+y_3^2-2 y_4^2+2 y_5^2, \\{} & {} \quad \lambda _2^2+\lambda _4^2-\lambda _2 y_1-\lambda _4 y_1-\lambda _2 y_2-\lambda _4 y_2+2 y_1 y_2-\lambda _2 y_3\\{} & {} \quad -\lambda _4 y_3+y_1 y_3+y_2 y_3-2 y_4^2-2 y_5^2\rangle . \end{aligned}$$

The other five associated primes are found by permuting indices of $\lambda _1,\lambda _2,\lambda _3,\lambda _4$. Hence, the Galois group $G_{\mathcal {L}}$ is the Klein four-group $S_2 \times S_2$ in $S_4$, and we infer the linear relation $\lambda _1+\lambda _2- \lambda _3-\lambda _4$. The set $E_3$ in step 7 is the singleton $\{z_1 z_2 - z_3 z_4\}$. The elimination in step 12 reveals the prime ideal in $\mathbb {R}[X]$ that is shown for arbitrary $d_1,d_2$ in Corollary 6.6.$\diamond$

Our final result is derived from Theorem 6.1 using tools of toric algebra [19, Chapter 8].

### Corollary 6.6

The Gibbs variety for QOT is parametrized by monomials $x_{ijkl} = y_{ik} z_{jl}$ that are not all distinct. Its prime ideal in $\mathbb {R}[X]$ is minimally generated by the $2 \times 2$ minors of a matrix of format $$\left( {\begin{array}{c}d_1+1\\ 2\end{array}}\right) \times \left( {\begin{array}{c}d_2+1\\ 2\end{array}}\right) $$, together with $$\left( {\begin{array}{c}d_1\\ 2\end{array}}\right) \left( {\begin{array}{c}d_2\\ 2\end{array}}\right) $$ linear forms in the entries of *X*.

We propose to extend QOT to quantum graphical models [30]. In statistics, every undirected graph *G* on *s* vertices defines such a model [27, Section 13.2]. The graphical model lives in the probability simplex $\Delta _{d_1 d_2 \cdots d_s-1}$. Its points are nonnegative tensors of format $d_1 \times d_2 \times \cdots \times d_s$ whose entries sum to 1. The quantum graphical model lives in the high-dimensional PSD cone ${\mathbb {S}}^{d_1 d_2 \cdots d_s}_+$, where the marginalization records the partial trace for every clique in *G*. It would be interesting to study the Gibbs manifold and the Gibbs varieties for these models. One may ask whether they agree for all graphs *G* that are decomposable. By Theorem 6.1, this holds for QOT, where *G* is the graph with two nodes and no edges.

## Data Availability

The datasets generated and/or analysed during the current study are available in the MATHREPO repository, https://mathrepo.mis.mpg.de/GibbsManifolds.
